# Modulation of event-related desynchronization in robot-assisted hand performance: brain oscillatory changes in active, passive and imagined movements

**DOI:** 10.1186/1743-0003-10-24

**Published:** 2013-02-26

**Authors:** Emanuela Formaggio, Silvia Francesca Storti, Ilaria Boscolo Galazzo, Marialuisa Gandolfi, Christian Geroin, Nicola Smania, Laura Spezia, Andreas Waldner, Antonio Fiaschi, Paolo Manganotti

**Affiliations:** 1 Department of Neurophysiology, IRCCS Fondazione Ospedale San Camillo, Venice, Italy; 2 Clinical Neurophysiology and Functional Neuroimaging Unit, Section of Neurology, Department of Neurological, Neuropsychological, Morphological and Movement Sciences, AOUI of Verona, Verona, Italy; 3 Neuromotor and Cognitive Rehabilitation Research Centre (CRRNC), USO Neurological Rehabilitation, Department of Neurological, Neuropsychological, Morphological and Movement Sciences, AOUI of Verona, Verona, Italy; 4 Department of Neurological Rehabilitation, Private Hospital Villa Melitta, Bolzano, Italy

**Keywords:** EEG, ERD, Active, Passive, Motor imagery, Bi-Manu-Track

## Abstract

**Background:**

Robot-assisted therapy in patients with neurological disease is an attempt to improve function in a moderate to severe hemiparetic arm. A better understanding of cortical modifications after robot-assisted training could aid in refining rehabilitation therapy protocols for stroke patients. Modifications of cortical activity in healthy subjects were evaluated during voluntary active movement, passive robot-assisted motor movement, and motor imagery tasks performed under unimanual and bimanual protocols.

**Methods:**

Twenty-one channel electroencephalography (EEG) was recorded with a video EEG system in 8 subjects. The subjects performed robot-assisted tasks using the Bi-Manu Track robot-assisted arm trainer. The motor paradigm was executed during one-day experimental sessions under eleven unimanual and bimanual protocols of active, passive and imaged movements. The event-related-synchronization/desynchronization (ERS/ERD) approach to the EEG data was applied to investigate where movement-related decreases in alpha and beta power were localized.

**Results:**

Voluntary active unilateral hand movement was observed to significantly activate the contralateral side; however, bilateral activation was noted in all subjects on both the unilateral and bilateral active tasks, as well as desynchronization of alpha and beta brain oscillations during the passive robot-assisted motor tasks. During active-passive movement when the right hand drove the left one, there was predominant activation in the contralateral side. Conversely, when the left hand drove the right one, activation was bilateral, especially in the alpha range. Finally, significant contralateral EEG desynchronization was observed during the unilateral task and bilateral ERD during the bimanual task.

**Conclusions:**

This study suggests new perspectives for the assessment of patients with neurological disease. The findings may be relevant for defining a baseline for future studies investigating the neural correlates of behavioral changes after robot-assisted training in stroke patients.

## Background

Robotic therapy in patients with neurological disease is an attempt to improve function in a moderate to severe hemiparetic arm. Robotic devices for upper limb rehabilitation in post-stroke patients include the MIT-Manus [1], MIME [2], NeReBot [3], and Bi-Manu-Track (BMT) robotic arm trainer [4-6]. Developed in parallel with robots for industrial applications, robotics in neurorehabilitation serve to treat the paretic upper limb after stroke [7]. The effects of training with the BMT, a robotic arm trainer that enables unilateral and bilateral passive and active practice of one degree of freedom pronation and supination movement of the forearm, as well as wrist dorsiflexion and volarflexion, were first investigated by Hesse in patients with sub-acute stroke and severe upper limb hemiparesis [5]. Stroke patients practiced 20 minutes every workday for six weeks using BMT-assisted bimanual active and passive movement of the forearm and wrist. Arm training with the BMT led to a greater improvement in upper limb motor control compared with the control group which had received only electrical muscle stimulation of the paretic wrist extensors.

Changes in cortical activity during active and passive movements and motor imagery in both normal subjects and stroke patients have been variously investigated using such different techniques as functional magnetic resonance imaging (fMRI) [8-13], positron emission tomography (PET) [14,15], magnetoencephalography (MEG) [16,17], near-infrared spectroscopy (NIRS) [18] and electroencephalography (EEG) [19]. Studies exploring the therapeutic utility of EEG have reported modulation in cortical activations during motor execution and imagery practices. In this context, EEG could be used to decipher thoughts or intent, so that a person could communicate with others or control devices directly by means of brain activity (brain computer interface) [20].

Functional brain activation related to movement preparation and execution is associated with a variety of event-related changes in EEG spectra. EEG oscillatory activity at 10-20 Hz over the premotor and primary sensorimotor areas (SM1), for example, typically decreases in power on motor tasks and produces the event-related desynchronization (ERD) phenomenon [19,21]. At the end of movement, rapid recovery of beta activity (beta synchronization), so-called event-related synchronization (ERS) [22-24], can also be observed over the ipsilateral side [24,25].

Research is sparse on cortical activity associated with robot-assisted therapy. To date, only two studies have reported cortical activity during robot-assisted tasks [26,27]. Using NIRS, Saeki et al. [27] investigated whether robotic training of the affected arm in a chronic stroke patient would lead to an increase in cortical activity in addition to evident motor recovery. The patient underwent robot-assisted training for 12 weeks with the BMT. During the active-passive mode training, asymmetrical activation was observed in the sensorimotor cortex, premotor cortex and supplementary motor area (SMA), but no regional activity was noted during bimanual passive movement. Mazzoleni et al. [26] evaluated the effects of robot-mediated therapy with the MIT-Manus on the upper limb in chronic hemiparetic subjects. They developed an integrated analysis of quantitative parameters computed from EEG signals, kinematic and dynamic data, and clinical assessment scales. Their preliminary results showed an improvement in upper limb motor ability and an increase in cortical activation, even one year after the acute event.

As demonstrated by simultaneous EEG-fMRI studies, voluntary movement can induce changes in oscillatory activity in the central areas underlying metabolic activation of sensorimotor areas [28,29]. Furthermore, a similar ERD distribution over the contralateral hand area can be observed during imagination of movement and during planning or preparation of a real movement [30-32]. Recently, in their study with combined EEG-fMRI, Formaggio et al. [33] reported a positive correlation between topographical changes in brain oscillatory activity and the blood oxygenation level dependent (BOLD) signal during a motor imagery task.

While active movement and motor imagery are well investigated, less attention has been focused on the effect of passive movement on brain activity [15,34-36]. Neuroimaging studies [15,37], under both active and passive conditions, detected metabolic activations in the SMA (stronger and more inferior than in the active condition) and in the inferior parietal cortex (on the convexity during active movements and in the depth of the central sulcus during passive movements). Neurophysiological studies applying the ERD approach [34,36] reported that during passive movement the beta ERD/ERS activity is similar in topography to that observed during voluntary movement without pre-movement components, suggesting that afferent proprioceptive inputs can play a role in brain oscillatory activity. The main limitation of studying passive movement is the lack of reliable standard devices that can induce and control the torque mechanism. The paucity of specific studies and the absence of a clear clinical or research paradigm of passive movement reflect the waning attention to this problem. With the recent and rapid development of robotic devices for training residual movement or to passively move plegic muscular segments, however, interest in the study of passive movement has been rekindled.

A better knowledge of cortical modifications after robotic therapy could inform the design and development of stroke rehabilitation protocols. An appreciation of these dynamics in cortical activation patterns during upper limb recovery relies on an understanding of the changes in motor control observed while the patient is executing a standardized well-controlled motor paradigm [38]. Building on the results from our previous EEG-fMRI studies [28,29,33], we used the same EEG analysis to investigate the topographical distribution of ERD/ERS during different robot-assisted tasks in healthy subjects. To do this, we evaluated the modifications of cortical activity during voluntary active movement, passive robot-assisted movement, and motor imagery performed under unimanual and bimanual protocols. The results may be relevant for defining a baseline in future studies on the neural correlates of behavioral changes after robot-assisted training in stroke patients.

## Methods

### Subjects

The study sample was 8 right-handed [39] healthy subjects (3 men and 5 women; mean age 26.38 years, standard deviation [SD] 2.62 years). All subjects gave written informed consent to participate in the study in accordance with the Declaration of Helsinki. The study design and protocol were approved by the Local Ethics Committee of the Verona University Department and Hospital.

### Experimental setup and motor paradigm

**Figure 1:**
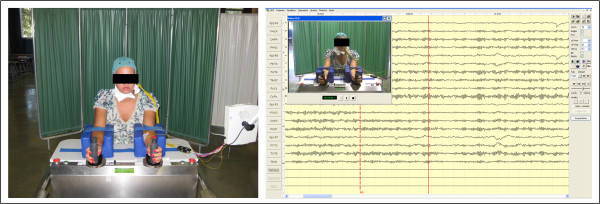
Bi-Manu-Track arm trainer and Video-EEG system.

Robot-assisted tasks were performed using the BMT robotic arm trainer (Reha-stim Co, Berlin, Germany) (Figure 1). The BMT consists of a height-adjustable table with two handles (3 cm in diameter) connected by an axis and linked to two respective electric motors. A computer controls the drives and records the data as amplitude, speed and resistance of movement. Two handle sets are available: one with a horizontal axis of rotation for the elbow and one with a vertical axis for wrist movement. To switch movement direction, the device is tilted 90° downward and the handles exchanged. A position control and the retroactive forces of the drive regulate the online recording of limb position and strength.

During the session the subject sat at a height-adjustable table with elbows bent at 90° and forearms in a mid position between pronation and supination in an arm trough (Figure 1). The BMT handle set has a vertical axis that enables wrist movement. The range of motion was set to 20° dorsiflexion and 20° volarflexion of the wrist. The speed was set at 60 repetitions of movement in 1 minute (1 Hz). The subject was asked to hold one or both handles depending on the motor paradigm.

The motor paradigm was performed during one-day experimental sessions composed of eleven protocols involving unimanual and bimanual active and passive movement and imagination of movement:

**Figure 2:**
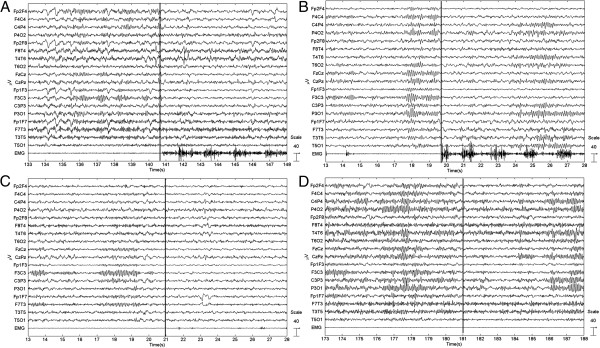
**EEG acquired during different tasks. A**) active movement with the right hand (subject no. 4), **B**) bimanual active movement (subject no. 2), **C**) passive movement with the right hand (subject no. 4), and **D**) imagination of movement with the right hand (subject no. 4). The vertical line represents the beginning of the task.

1. active movement with the right hand (Figure 2A);

2. active movement with the left hand;

3. bimanual active movement (Figure 2B);

4. passive movement with the right hand (right hand moved by the BMT) (Figure 2C);

5. passive movement with the left hand (left hand moved by the BMT);

6. bimanual passive movement (both hands moved by the BMT);

7. active – passive movements (the right hand drives the left hand in a mirror-like fashion);

8. active – passive movements (the left hand drives the right hand in a mirror-like fashion);

9. imagination of movement with the right hand (Figure 2D);

10. imagination of movement with the left hand;

11. imagination of bimanual movement.

**Figure 3:**
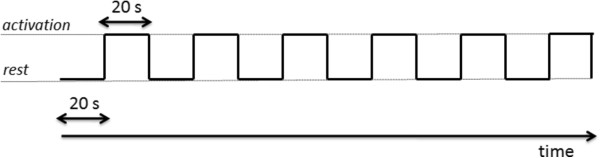
**Time schedule for measuring EEG data during a task.** Six runs of rest alternating with six runs of execution.

The protocols were delivered in random order across the subjects. Six runs of rest alternating with six runs of execution were performed in each session (each run lasted 20 s) (Figure 3). Task execution was acoustically paced with a metronome at a frequency of 1 Hz. The metronome ticking continued during activation and rest blocks to keep the sensory input constant; the subjects were signaled to start and stop the task when the experimenter gave the instruction “start” and “stop”, respectively. To perform the task correctly, each subject was trained for several minutes before the experiment.

The electromyographic (EMG) signal, recorded from the right and left flexor muscles of the forearm with Ag/AgCl surface electrodes fixed on the skin with a belly-tendon montage, was acquired to monitor movements required by the tasks, as well as involuntary mirror movements or other unspecific muscle activations. EEG and video were recorded synchronously.

During the active and passive movement tasks, the subjects were instructed to keep their eyes open, to avoid blinking, and to look at a stationary point near a camera positioned 2 m away from them. Before each recording, the subjects were given a moment to focus their attention. The eyes-open condition was chosen in order to minimize contamination by the posterior alpha rhythm, since both rhythms (occipital and Rolandic) fall within the same frequency range. During the motor imagery task, the subjects were instructed to keep their eyes closed so that they could better “feel” the movement. They were asked to imagine the kinesthetic experience of movement without tensing their muscles.

### EEG data acquisition

The EEG data were acquired using a video EEG system (Ates Medica Device, Verona, Italy) and a cap (SEI EMG s.r.l, Padova, Italy) providing 21 Ag/AgCl electrodes positioned according to a 10/20 system (impedance was kept below 10 kΩ) and two surface electrodes to acquire the EMG signal (Figure 1). The reference was placed anterior to Fz and the ground posterior to Pz. The EEG data were acquired at a rate of 250 Hz using the software package Geodesic EEG System on Neurotravel technology (Ates Medica Device, and Electrical Geodesic, Inc., Eugene, OR).

### EEG data analysis

The data were processed in Matlab 7 (MathWorks, Natick, MA) using scripts based on EEGLAB 4.51 (EEGLAB toolbox for single trial data analysis, Swartz Center for Computational Neurosciences, La Jolla, CA; http://www.sccn.ucsd.edu/eeglab), as well as a dedicated home-made code created for this study. Visible artifacts in the EEG recordings (i.e., eye movements, cardiac activity, and scalp muscle contraction) were removed using an independent component analysis (ICA) procedure [40].

The data were processed using an average reference. The EEG recordings were band-pass filtered from 1 to 30 Hz using a finite impulse response (FIR) filter. The EEG data of each rest and active run (lasting for 20 s) were divided into 9 epochs of 2 s. A fast Fourier transform (FFT) was applied to non-overlapping epochs, each containing 500 data points for all the electrodes and for the two experimental conditions, and then averaged across epochs under the same conditions. The recordings were Hamming-windowed to control for spectral leakage. Power spectra density *P*_*x*_(*f*) (μV^2^/Hz) was estimated for all frequencies between 0 and 125 Hz. Because movement preparation and execution produce ERD over the sensorimotor area at 10 and 20 Hz [41], only the upper alpha (10–12 Hz) and beta (13–30 Hz) frequency ranges were analyzed. An accepted ERD/ERS procedure was used to quantify the event-related relative changes in EEG power at an electrode *x*[42,43] according to Eq. 1:

$$\text{ER}D_{x}\left(f\right)=\left(P_{\text{xactivation}}\left(f\right)-P_{\text{xrest}}\left(f\right)\right)/P_{\text{xrest}}\left(f\right)\times 100.$$

The ERD/ERS transformation was defined as the percentage decrease/increase of instant power density at the ”event” compared with a “pre-event” baseline value. Event-related power decreases (“cortical activation state”) that implied a decrease in synchrony of the underlying neuronal populations were therefore expressed as negative values, whereas event-related power increases (“cortical idling state”) were expressed as positive values. A topographic map showing the changes in ERD/ERS for each subject for the alpha and beta ranges and a grand mean map for all the subjects were computed.

A paired sample two-tailed t-test was computed to identify significant differences between ERD/ERS values in the alpha and beta ranges and a reference condition. Then, two-dimensional grand mean t-maps of ERD were computed from the t-values to check the topographical distribution of the significance [28]. ERD/ERS t-maps were thresholded at p<0.05 (|t|>2.306).

## Results

### Group EEG analysis

#### Active movement

**Figure 4:**
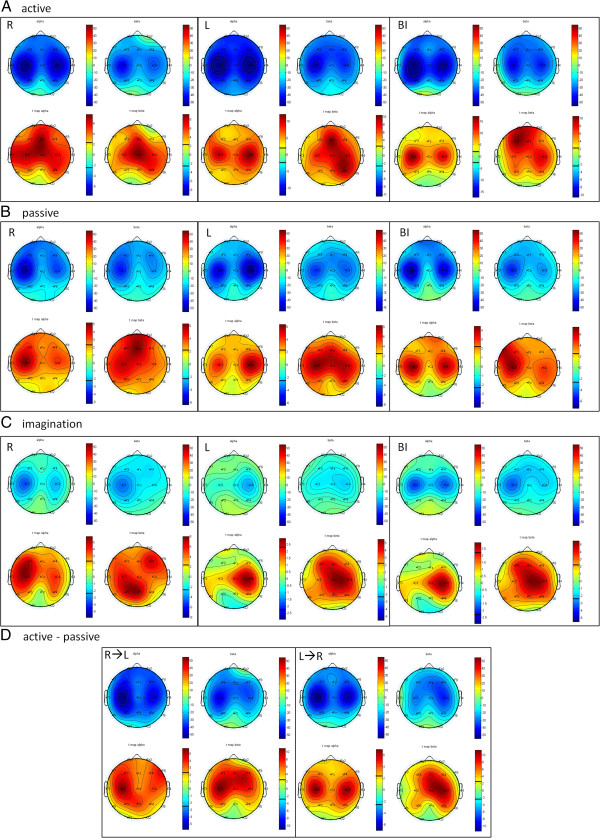
**Topographic maps showing ERD and t values.** Grand average maps of ERD/ERS in alpha and beta bands during active (**A**) and passive (**B**) movements, imagination of movement (**C**), active-passive movement (**D**) (R: right, L: left, BI: bimanual). Blue color coding indicates maximal ERD. T-maps of ERD/ERS in alpha and beta bands thresholded at p<0.05 (|t|>2.306).

During right hand movement, the mean alpha and beta maps showed a decrease in ERD over the central SM1 areas, contralateral and ipsilateral to the movement (C3 and C4), in a balanced way in the alpha range and with predominance on C3 in the beta range. The t-maps showed significant changes (p<0.05) in alpha desynchronization bilaterally over SM1 and over the fronto-central area (Fz, Cz); in the beta band, these changes were localized only over Cz (Figure 4A-left).

During left hand movement, the mean alpha and beta maps showed a decrease in ERD over the central SM1 areas, contralateral and ipsilateral to the movement (C3 and C4) similarly to the maps obtained during right hand movement. The t-maps showed a significant desynchronization (p<0.05) over C3 and C4 in the alpha range; in the beta band, ERD was observed over the contralateral SM1 (C4, P4) and over the SMA (electrodes Fz, Cz) (Figure 4A-center).

Bimanual active movement produced alpha ERD in C3 and C4, much more localized over the left SM1, and significant beta ERD in C4 and over the left frontal area (F3, Fz, Fp1), as noted on the statistical t-maps (Figure 4A-right).

### Robot-assisted passive movement

Right hand passive movement produced desynchronization over C3 and C4; the t-maps showed significant ERD (p<0.05) over the contralateral SM1 (C3) in both the alpha and beta bands, with a decrease in beta rhythm also over the frontal electrodes (Fz) (Figure 4B-left).

Left hand passive movement produced localized ERD bilaterally, with predominance on the contralateral side in the alpha range. The t-maps showed significant ERD (p<0.05) in the alpha range over the contralateral SM1 (C4) and over C3, Fz and C4 in the beta range (Figure 4B-center).

During bimanual passive movement there was bilateral desynchronization; the t-maps showed significant ERD (p<0.05) over C3 and C4 in the alpha range and more lateral desyncronization (C3) in the beta range (Figure 4B-right).

### Imagination of movement

During imagination of right hand movement, localized ERD was noted over the contralateral SM1. This phenomenon was prominent (t-maps) over C3 in the alpha range and over the contralateral parietal electrodes (P3, Pz) in the beta range (Figure 4C-left).

During imagination of left hand movement, desynchro-nization over C4 was observed. The t-maps showed significant ERD (p<0.05) over C4 in both the alpha and beta bands, with a significant decrease in Cz in the beta range (Figure 4C-center).

During bimanual movement imagination, alpha ERD was localized bilaterally, more prominently over C4; significant beta ERD (p<0.05) was noted over C4 and Cz (Figure 4C-right).

### Active-passive movements

When the right hand drove the left one, the alpha ERD changes were localized bilaterally, while beta ERD was prevalent over C3. Alpha ERD was significantly localized (p<0.05) over the left SM1 area (C3) and beta ERD over the SM1c and over the fronto-central electrodes. When the left hand drove the right one, desynchronization was significantly localized (p<0.05) bilaterally over SM1 in the alpha band and over Cz-C4 in the beta range (Figure 4D).

### Individual EEG analysis

All subjects activated both sensorimotor areas in the alpha range during unilateral active movement; bilateral activation was more evident on the right hand task, while unilateral desynchronization was observed in the beta range. Bimanual active movement produced alpha and beta ERD in both SM1 areas in all subjects.

In three subjects, right hand passive movement produced unilateral ERD over SM1c, while left hand passive movement produced bilateral activation in the alpha and beta ranges. Bilateral activation during right and left passive movement was observed in the other four subjects, where beta ERD was more localized over SM1c. One subject showed ERD over SM1c during left hand passive movement and over both SM1 areas during right hand passive movement. Bilateral activation over the sensorimotor area during bimanual passive movement was observed in all subjects.

During imagination of movement with the right hand, alpha ERD was localized bilaterally and beta ERD over SM1c in all subjects except one, where unilateral desynchronization was also observed in the alpha range. ERD localization during imagination of movement with the left hand differed among subjects: alpha and beta ERD were evident over both SM1 areas in 2/8 subjects (nos. 1 and 8); alpha ERD over SM1c and bilateral beta ERD were noted in 3/8 subjects (nos. 3, 5, and 7); alpha ERD was localized bilaterally and beta ERD unilaterally in 2/8 subjects (nos. 2 and 4); finally, ERS in the alpha range over both SM1 and ERD in the beta range over SM1c were noted in 1/8 subjects (no. 6). Bilateral activation over the sensorimotor area in the alpha range was observed during bimanual imagination of movement in all subjects, while beta ERD was more lateralized.

When the right hand drove the left one, the alpha ERD changes were predominantly localized over the left SM1 area (C3) and the beta ERD over both SM1 and over Cz in 3/8 subjects (nos. 1, 2, and 6); localization was bilateral in both ranges in the other subjects. When the left hand drove the right one, desynchronization was predominantly localized bilaterally over SM1 in the alpha and beta ranges and also over Fz, Cz in the beta range in 2/8 subjects (nos. 4, and 7).

## Discussion

This study reports for the first time a neurophysiological assessment of changes in cortical activity during different robot-assisted tasks. ERD-ERS analysis showed bilateral activation of SM1 during unilateral movement, albeit with predominant contralateral activation, whereas the activations were localized over the SM1c during passive unilateral movement and the imagination of unilateral movement. The alpha and beta t-maps are not superimposable; indeed, beta activation is more anterior, corresponding to the SMA. During all bimanual movements significant ERD was noted bilaterally over SM1 (C3 and C4).

The new main finding of the study is the significant desynchronization of alpha and beta brain oscillations during passive robot-assisted motor performance. Unilateral passive movement induced localized ERD over the contralateral SM1 area, with a scalp topography similar and even more localized than the ERD produced during performance on the active motor tasks. The ERD during passive robot-assisted movements was consistent and reliable across all eight subjects in both frequency bands, albeit with small topographical differences (more anterior in the beta band, more posterior in the alpha band). Furthermore, bimanual passive robot-assisted movement induced significant bilateral ERD over the sensorimotor areas.

Published data on passive movement are discordant and not enthusiastic. Pfurtscheller and Aranibar [19] reported a clearer and more consistent variation in ERD and ERS during active movement than during passive movement. Their study involved mostly patients with deafferentation problems in order to exclude proprioceptive input on motor activation. Alegre et al. [34] reported that beta ERD/ERS during passive movements was similar in topography to that observed during voluntary movements, but without pre-movement components. Significant ERD over the contralateral M1 during active movement and during passive movements induced by functional electrical stimulation (FES) were reported in a recent EEG study [35], but passive FES did not produce observable pre-motor ERD during an active motor task. In a [^15^O]H_2_O PET study, Weiller et al. [15] compared voluntary movements of the right elbow with passive movements driven by a torque mechanism in healthy subjects. Postcentral gyrus activation was almost identical in the primary sensorimotor cortex during both passive and active movements; SMA activation was also observed.

The lack of reliable and standard devices to induce and control the torque mechanism is one of the main limitations of studying passive movement. Added to this problem is that in most cases these devices are incompatible with the use of MRI. Instead, the EEG technique has considerable advantages over other methods for studying passive movements. PET, SPECT and fMRI are inconvenient since they require positioning the subject on the scanner bed inside the tube; because of these physical constraints, subjects cannot perform particular movement tasks. Moreover, since the BMT machine is not MR compatible, it cannot be used inside the MR room.

One way to obviate such obstacles is with EEG. The correlation between EEG and fMRI documented in recent studies [28,29,33] reinforces its reliability as a noninvasive parameter of brain activation and fits well with use of the robotic device. In this study, by combining EEG with the BMT we were able to noninvasively detect the effects of robot-assisted movements on brain oscillatory activity. In so doing, we were also able to control motor execution with prefixed performance parameters of velocity, degree of angular movement and frequency of complex intra/extra rotation of hand movement. These findings may inform future applications of passive robot-assisted movement in rehabilitation therapy.

A second main finding is that hand movement markedly activates the contralateral side, albeit with prominent bilateral activation on both unilateral and bilateral active motor tasks in all subjects. This result is in line with previous observations. In their fMRI study, Newton et al. studied BOLD activation during hand movement in six subjects and observed significant inhibition of the ipsilteral side and activation of the contralateral side. Significant BOLD signal decreases were observed in the ipsilateral M1. This finding appears consistent with the interhemispheric interactions that occur between the M1 of each hemisphere and increased neuronal activation in M1 of the opposite hemisphere [9]. Differently, it was observed that unilateral movement produces ipsilateral activation as well, particularly when movement is performed with the dominant arm [18].

It is also known that activations occur in the right and left motor cortex, pre-SMA, premotor cortex, prefrontal cortex, bilateral somatosensory cortex, and parietal cortex along the intraparietal sulcus, suggesting an influence of somatosensory processes in bimanual movement control, as found in right-handers [44-49]. Bai et al. [50], for instance, investigated spatiotemporal features of hemispheric asymmetry by quantifying ERD/ERS before and after a complex motor task of self-paced sequential finger movements performed on either left or right side. They found that a difference of ERD distribution between left and right hand movements was only observed during motor preparation: bilateral ERD for left movements and contralateral ERD for right hand movement, suggesting that hemispheric asymmetry might be a property of neural organization during motor preparation. In order to determine whether or not there is functional asymmetry in motor areas, regional cerebral blood flow (rCBF) was measured with PET in healthy subjects in order to compare rCBF changes related to movements of the dominant (right) and the non-dominant (left) hand [51]. Movements of the dominant hand and the non-dominant hand increased CBF in the contralateral motor area and the premotor area, with small increases in the supplementary motor area. However, movements of the non-dominant hand also elicited significant ipsilateral increases. rCBF changes in the motor areas and the prefrontal area of one hemisphere are not related simply to movement of the contralateral hand. Non-dominant hand movement may also require activation of the ipsilateral side, suggesting asymmetry of function in human motor cortical areas.

A possible explanation for the bilateral activation during unilateral movement observed in our study could be sought in the type of the movement performed. For example, flexor-extension of the wrist can be viewed as a more proximal task than movement of the fingers, which has a more bilateral activation. The role of the wrist in upper limb movement can be considered postural to the extent that it stabilizes the wrist joint and allows the fingers to move [52]. Bilateral activation could be due to the complexity of movement in relation to wrist flexion and the grasping of fingers over the device (joystick); bilateral activation can increase from simple to more complex performance also for distal finger movement, which is associated with an increase in event-related coherence between the two homologous areas [53].

A third new finding of this work is the effects on brain oscillatory activity of active-passive movements. When the right arm drives the left one, a predominant activation in the contralateral side (SM1c) can be observed. Conversely, when the left arm drives the right one, activation is bilateral, especially in the alpha range. This type of movement was studied by Saeki et al. using NIRS. They found no significant activation during bimanual passive movement and an activation in bilateral M1 and SMA during active-passive movement (non-affected arm drives affected arm) [27].

Finally, during the motor imagery tasks the t-maps showed contralateral ERD on the unilateral task and bilateral ERD on the bimanual task. These findings are in line with our previous observations [33] where, using EEG and fMRI, we found a decrease in ERD localized over the SM1c (during imagination of movement of the right hand) and slight desynchronization in the ipsilateral side (SM1i), whereas the mean beta map showed a decrease in ERD over the SMA. fMRI showed significant activation in the SMA, SM1c, SM1i, and in the ipsilateral cerebellum. The correlation was negative for the contralateral side and positive for the ipsilateral side. The localization of these activations was similar to that obtained during active hand movement, but the power spectra and the ERD values were lower. These findings support the idea reported elsewhere [10,31] that active and kinesthetic experiences of movement share the same functional networks activated during movement planning, preparation and execution. A similar ERD distribution over the contralateral hand area can be observed during imagination of movement and during planning or preparation of a real movement [32], but the activity in this area is typically much greater during motor execution than during motor imagery.

## Conclusions

This study suggests new perspectives for neurological assessment by evaluating cortical oscillatory activity in stroke patients presenting with either motor or sensitive deficit due to lesions of different systems involved in motor control or also without motor deficit, e.g., patients with aphasia or neglect. In such patients, the reorganization phenomena of the motor cortex before and after training with various different robotic devices would be interesting to compare.

The major novel aspect of the present study is the evaluation of cortical activity generated by movements in highly standardized robot-assisted paradigms. The technique has proven very effective in determining variations in cortical activity during various types of movements. Nonetheless, despite advances in rehabilitation strategies, the neural mechanisms underlying functional recovery remain elusive. Currently, it is unclear whether the observed cortical reorganization is due to spontaneous recovery processes or to rehabilitation. Also unclear in the majority of treatment procedures where motor recovery is investigated are the effects different types of exercises (active and passive movements) can exert on the central nervous system. What is clear is that future study is needed to further our understanding of the mechanisms underlying motor recovery and inform the development of a new clinical approach to upper limb rehabilitation in stroke patients.

## Competing interests

The authors declare that they have no competing interests.

## Authors’ contributions

EF carried out the studies, data acquisition, analysis and interpretation, drafted the manuscript, and performed the statistical analysis. SFS and IBG carried out the studies, data acquisition, analysis and interpretation, drafted the manuscript. MG and CG carried out the studies, data acquisition and interpretation, drafted the manuscript. LS carried out the studies and data acquisition. AW assisted in drafting the manuscript. AF, NS and PM conceived of the study, participated in its design and coordination, and assisted in drafting the manuscript. All authors read and approved the final manuscript.
